# Microcomputed Tomographic Assessment of the Single Cone Root Canal Fillings Performed by Undergraduate Student, Postgraduate Student and Specialist Endodontist

**DOI:** 10.3390/jcm10051080

**Published:** 2021-03-05

**Authors:** Saulius Drukteinis, Goda Bilvinaite, Paulius Tusas, Hagay Shemesh, Vytaute Peciuliene

**Affiliations:** 1 Institute of Dentistry, Faculty of Medicine, Vilnius University, Zalgirio 115, LT-08217 Vilnius, Lithuania; goda.bilvinaite@gmail.com (G.B.); paulius.tusas@gmail.com (P.T.); vytaute.peciuliene@mf.vu.lt (V.P.); 2 Academic Centre for Dentistry Amsterdam (ACTA), Gustav Mahlerlaan 3044, 1081 LA Amsterdam, The Netherlands; H.Shemesh@acta.nl

**Keywords:** single cone, BioRoot RCS, micro-computed tomography, undergraduate student, postgraduate student, endodontist, porosity

## Abstract

The present study evaluated the obturation quality of root canals filled with BioRoot RCS sealer and single gutta-percha point by undergraduate student (US), postgraduate student (PS) and endodontist (ED). Twenty-one plastic models of upper premolars were enlarged with HyFlex EDM instruments to a size 40/0.04 taper and randomly divided into three groups (7 teeth/14 canals per group): US, PS and ED. After the obturation of root canals with BioRoot RCS and one HyFlex EDM size 40 gutta-percha point, plastic models were scanned using micro-computed tomography scanner (µCT) SkyScan 1272 at isotropic resolution of 10 µm. The porosity distribution was evaluated separately for the apical, middle and coronal thirds. The Kruskal–Wallis, Mann–Whitney, Friedman and Wilcoxon tests with the significance level set at 5% were used for data analysis. The µCT evaluation revealed open pores being the dominant type of porosity in all experimental groups and root canal thirds, with the highest percentage of pores in the apical third of root canal fillings. The quality and homogeneity of single cone root canals fillings remained similar between the groups in the apical and middle thirds (*p* > 0.05). Significant differences were observed only in the coronal third (*p* < 0.05).

## 1. Introduction

The quality of root canal obturation plays an essential role to ensure the successful long term outcome of endodontic treatment [1]. Studies show that approximately 60% of endodontic treatment failures can be associated with a poor root canal obturation [2]. Root canals are considered properly obturated if the filling material is uniformly homogeneous over the entire root canal working length (WL) and has appropriate conical taper resembling the root canal’s internal shape after preparation [3]. High-quality hermetical sealing of the root canal system prevents the obturated root canals from recontamination and penetration of microorganisms and their metabolites into periapical tissues [4]. It has been shown that the homogeneity of the filling is directly related to the voids and pores inside the material [5]. Therefore, the porosity of the material can be used as one of the objective criteria for assessing the quality of the root canal obturation. There are two types of pores identified in root canal fillings: closed (internal) or open (external) pores [6]. Closed pores are surrounded by the filling material and have no contact with the root canal walls, making them clinically less significant for endodontic treatment outcomes [7]. Meanwhile, the open pores formed between the filler material and the canal walls can have a substantial clinical impact [8]. The network of open pores can be an excellent pathway for microleakage and possible worse clinical outcome [9].

Various materials and filling techniques were suggested for the root canal obturation to achieve hermetic, voids free fillings and prevent possible microleakage [10]. However, it has been demonstrated that there is no technique for root canal obturation, which can ensure the impeccable three-dimensional sealability of the root canal system [11,12]. It has been shown that the highest percentages of voids as well as poorer adaptation of the material to the root canal walls and irregularities are detected in the fillings when the cold lateral compaction technique was used for obturation [11,13]. One of the main reasons for the high porosity of the laterally compacted gutta-percha/sealer fillings may be the insufficiently deep insertion of the auxiliary gutta-percha points into space previously made by the spreader [14]. Some studies demonstrated no advantages of the thermoplastic gutta-percha obturation techniques over cold lateral compaction in terms of porosity of the fillings, while the other studies claim that thermoplastic techniques ensure less porous fillings [15,16]. However, one of the main reasons that can lead to the formation of pores and voids in the mass of the softened gutta-percha is shrinking of the material when it cools down [17]. Besides, it should be highlighted that the lateral compaction technique is quite complicated for operator and time-consuming, while the thermoplastic obturation techniques additionally require expensive armamentarium and devices for obturation [2,10].

The simplified single cone (SC) root canal obturation technique, when a single gutta-percha point in conjunction with hydraulic calcium silicate-based sealer is used to fill the root canals, is rapidly gaining popularity among the clinicians [18]. These sealers are highly biocompatible and bioactive, possess antibacterial activity, low solubility, shrinkage and long-term dimensional stability, enabling to use of a higher volume of the sealer in conjunction with tapered gutta-percha points [7,8,14]. In contrast to the previously discussed obturation methods, the SC obturation technique is simple to use, does not require a long learning curve, is clinically appealing and does not require any additional armamentarium or devices. During the obturation, no lateral or vertical compaction is used, therefore minimizing the risks to provoke dentinal defects or cracks [19]. Numerous studies have demonstrated that the amount of the overall porosity of the SC fillings is comparable to other techniques or even less [12,20,21]. Moreover, the hydraulic calcium silicate-based sealers used with a SC technique have enhanced adhesion to the root canal dentin in comparison to other types of the sealers and ensure a dimensionally stable, more tight and hermetic seal [22,23]. Additionally, due to the superior flowability, hydrophilicity and the small particle size of the material, the excellent penetrability of the sealers can be achieved even without any condensation pressure [19]. Recent clinical studies demonstrated the high clinical success rates when the SC technique was used for root canal obturation in cases of primary endodontic treatment or endodontic retreatment [24,25].

It has been concluded that the success rates of endodontic treatment highly depend on the quality of root canal shaping, cleaning and eradication of the microorganisms [1,2,14]. Moreover, the impact of the quality of the root canal obturation and the clinical experience of the operator has also been demonstrated [9,10,25]. The clinical success rates when treatment procedures are performed by a specialist endodontist can reach more than 90% or 95%, whereas in cases where general practitioners perform root canal treatment, the probability of the clinical success can decrease even to 40–65% [26,27,28,29]. Moreover, even lower success rate is observed when endodontic treatment is completed by undergraduate students [30]. Eskandarloo et al. (2017) demonstrated that the quality of root canal obturation, detected on the periapical radiographs, performed by dental students is commonly poor and unacceptable [31]. Other investigations revealed that only 30.1–47% of the root canals obturated by undergraduate dental students are under acceptable quality standards [27,32]. Therefore, the better obturation quality was achieved by students in a postgraduate endodontic program in comparison to undergraduates [27]. However, it has been claimed that the recently introduced a simplified single cone root canal obturation technique potentially can be less related to the clinical experience of the operator [12,33].

Microcomputed tomography (µCT) is a widely accepted, non-destructive method for the two-dimensional (2D) and three-dimensional (3D) assessment of the root canal fillings [12,33]. µCT imaging enables to assess the overall porosity of the material, porosity distribution, identify the type of the pores and using the specific software calculate volumetric values and parameters or visualize in 3D [8,15,17]. Despite the simplicity and clinical appeal of the SC obturation technique, there is no information on how the clinical experience of the operator can determine the root canal obturation quality and porosity of these fillings. Therefore, the aim of this study was, by means of µCT analysis, to assess and compare the porosity distribution in the single gutta-percha cone/hydraulic calcium silicate-based sealer fillings performed by under- and postgraduate students and specialist endodontist. The null hypothesis tested was that the clinical experience of the operator does not have any impact on the quality of the root canal obturation.

## 2. Materials and Methods

### 2.1. Specimen Preparation

Twenty-one standardized 3D plastic models of upper premolars (DRSK, Hassleholm, Sweden) with pre-opened endodontic accesses were used for this study. The plastic teeth had two separate roots and Type I canal configuration according to the Weine’s classification. The working length (WL) was determined by inserting a size 10 K-file (Dentsply Maillefer, Ballaiques, Switzerland) into the root canal until the tip of the instrument was visible at the apical foramen. The WL was established 1 mm short of the apex. Root canals were subsequently shaped with HyFlex EDM (Coltene, Langenau, Germany) rotary NiTi endodontic files at the rotation speed of 400 rpm and the torque of 2.5 Ncm, powered by X-Smart (Dentsply Sirona, Ballaiques, Switzerland) endodontic motor. Instruments were used to the full working length in the following sequence: Glide-path file (size 10/0.05 taper), Preparation file (size 20/0.05 taper), OneFile (size 25/~taper) and Finishing file (size 40/0.04 taper).

The root canals were repeatedly irrigated with 5 mL 3% sodium hypochlorite (Ultradent Products Inc., South Jordan, UT, USA) after each instrument. At the end of preparation, 5 mL of 17% ethylenediaminetetraacetic acid (Ultradent Products Inc., South Jordan, UT, USA) was used for 2 min. 5 mL of sterile distilled water was used as a final flush. Irrigation was performed using disposable syringes and 29-G NaviTip needles (Ultradent Products Inc., South Jordan, UT, USA). After irrigation, root canals were dried with size 40/0.04 taper paper points (Coltene, Langenau, Germany). All specimens were prepared by a single operator–specialist endodontist.

### 2.2. Root Canal Obturation

The sample size of experimental groups was calculated using G*Power 3.1 software (Heinrich Heine, Iniversität Düsseldorf, Düsseldorf, Germany) following t-test family and the difference of two independent means with alpha error probability of 0.05, and a power (1-beta error probability) of 0.95. Therefore, a total of 12 root canals was indicated as the minimum required sample size. After preparation plastic models were fixed into prefabricated A-silicone (3M Express, 3M ESPE, Seefeld, Germany) blocks up to the cemento-enamel junction to ensure the blindness of the root canal filling procedure. Specimens were randomly divided into three experimental groups (7 teeth per group), according to the operator performing root canal obturation: undergraduate student (US), postgraduate student (PS) and endodontist (ED). The SC obturation technique was theoretically introduced to the fourth-year undergraduate student by experienced academician and specialist endodontist before root canal obturation by giving a 1-h presentation, following the 2 h hands-on practical training. The third-year postgraduate student in the endodontology program was already familiar with SC obturation technique and has been used in daily clinical practice for three years. Finally, the root canals in the ED group were obturated by endodontist with an over fifteen years of clinical experience.

A total of 14 canals in each group were filled with BioRoot RCS (*Septodont*, Saint-Maur-des-Fosses, France) and one HyFlex EDM size 40.04 gutta-percha point (Coltene, Langenau, Germany). The pre-fitted gutta-percha point was inserted into the root canal with a tug-back effect at the WL. The cone was coated with a small amount of the sealer mixed according to the manufacturer’s instructions and slowly inserted into the root canal to coat the root canal walls. The procedure was repeated twice to deliver the required amount of the sealer into the root canal. Finally, the last third time gutta-percha cone was recoated with the sealer and gently inserted to the full working length. The gutta-percha cone was subsequently cut with a heat carrier at the level of the orifice. The endodontic accesses were filled with temporary filling material (Cavit™-W; 3M ESPE, Seefeld, Germany) and submerged into the thermal bath (Thermo Scientific™ Precision™; Fisher Scientific; Vantaa, Finland) containing 37 °C water for 1 week to allow the filling material set completely before further analysis.

### 2.3. Micro-CT Scanning and Analysis

A high-resolution micro-CT scanner SkyScan 1272 (Bruker, Kontich, Belgium) was used to scan the specimens. All scans were performed using a 90 kV source voltage, 111 µA beam current, 10 µm isotropic resolution, 0.2° rotation step and 1350 ms exposure time. A 0.5 mm aluminum and 0.038 mm copper filter was used for artefact reduction. Images obtained from the scan were reconstructed using NRecon v.1.7.1.0 software (Bruker, Kontich, Belgium) with a ring artefact reduction factor of 3 and beam hardening correction of 20%.

The reconstructed images were analyzed using CTAn v.1.14.4.1 software (Bruker, Kontich, Belgium). After the selection of root canal contours, the gray scale range required to recognize the filling materials and voids was determined in a density histogram by using a global threshold method. Comparisons between the original and segmented scans were performed to ensure segmentation accuracy. The volume of root canal was selected as the volume of interest (VOI) and subsequently the voids (VVol), filling material (FVol), open pores (OPVol) and closed pores (CPVol) was determined by processing the segmented images with a custom-processing tool. The percentage volume of open pores (%OPVol) and closed pores (%CPVol) was calculated using the following formulas:

% OPVol = OPVol/(VVol + FVol) × 100

%CPVol = CPVol/(VVol + FVol) × 100

All images were examined by a single evaluator who was blinded to data regarding experimental groups and their specimens. The percentage volume of open and closed pores was calculated separately for the apical, middle and coronal thirds at intervals of 3 mm of each third, and a total VOI of 9 mm was selected for assessment. The last apical 1 mm of the root was not included in the analysis. The CTVol v.2.2.3.0 software (Bruker, Kontich, Belgium) was used for 3D volumetric visualization and qualitative evaluation of the fillings.

### 2.4. Statistical Analysis

Statistical analysis was performed using SPSS 25.0 software (SPSS Inc., Chicago, IL, USA). The Shapiro–Wilk test revealed a non-normal distribution of the data. Therefore, the differences among experimental groups were compared using a non-parametric Kruskal–Wallis test. When statistically significant *p*-values were found, the Mann–Whitney test was applied for pairwise comparisons. The differences between the root canal thirds in the same group were determined using a non-parametric Friedman test followed by the Wilcoxon test for pairwise comparisons. The significance level was set at *p* < 0.05.

## 3. Results

**Figure 1:**
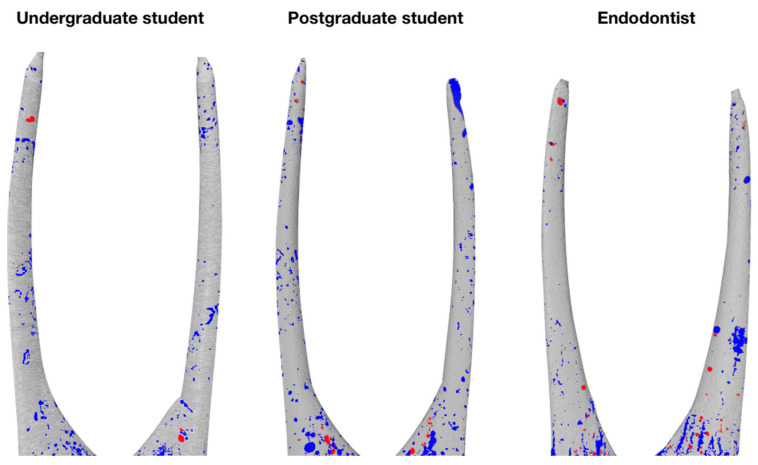
Three-dimensional reconstructions of the single cone root canal fillings of different experimental groups, demonstrating distribution of open (blue) and closed (red) pores.

The micro-CT evaluation revealed pores of various diameter and shape inside the mass of the hydraulic calcium silicate-based sealer as well as in the interface of the sealer and root canal walls, while the open pores were predominant type of porosity in all groups and thirds evaluated (Figure 1). No pores inside the gutta-percha points were detected as might have been expected.

The results of the volumetric analysis of the porosity of root canal fillings are detailed in Table 1. The distribution of open and closed pores demonstrated statistically significant differences among all groups in the coronal third (*p* = 0.017 and *p* = 0.02, respectively). Regarding the results of pairwise comparisons, the percentage of open pores did not differ significantly only between PS and ED groups, closed pores—between US and PS groups (*p* > 0.05). In the middle third all groups exhibited similar amount of open pores (*p* = 0.06), however, the distribution of closed pores remained significantly different (*p* = 0.014). Pairwise comparisons revealed that the percentage of closed pores did not differ significantly only between US and PS groups (*p* > 0.05). The apical third of root canal fillings had no statistically significant differences by comparing the amount of both open (*p* = 0.56) and closed (*p* = 0.12) pores.

**Figure 2:**
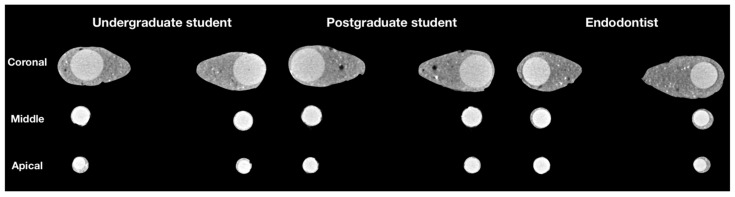
2D cross-sectional images at the different thirds of the obturated root canals of random samples of US, PS and ED groups, demonstrating various porosity inside the fillings.

The cross-sectional 2D images of the three experimental groups at the different root canal thirds are shown in Figure 2. The micro-CT analysis revealed that all groups exhibited the highest percentage of open and closed pores in the apical third of root canal fillings. Furthermore, statistically significant differences were observed in the distribution of open and closed pores within the same group when comparing all root canal thirds (*p* < 0.05).

Regarding the results of pairwise comparisons, which are summarized in Table 2, no statistically significant differences were detected only between coronal and middle thirds, where the percentage of open pores remained similar in all experimental groups (*p* > 0.05).

## 4. Discussion

It has been demonstrated that the porosity of root canal fillings is directly associated with an unfavourable endodontic treatment outcome [34]. The pores inside the mass of the materials can adversely affect their physical properties and create favourable conditions for the microleakage [35]. For these reasons, porosity is considered to be one of the main criteria for assessing the quality of root canal fillings [11,36]. Therefore, in our study, the porosity of the fillings was assessed and compared to determine the quality of root canal obturation using the SC technique performed by the operators with a different clinical experience.

Different methods to evaluate the porosity and microleakage of the fillings, such as bacterial, glucose, radioactive isotope, or dye penetration tests, have been used with their significant limitations [37]. Recently, the microcomputed tomography was considered as the optimal, non-destructive method allowing quantify and qualify the high-resolution images in 2D and 3D [8,12,33]. Therefore, in our study, the assessment of the porosity of the SC fillings was performed using µCT imaging. However, it should be mentioned that some drawbacks of the technique should be considered. Despite the high accuracy of µCT, tiny pores may still be undetectable due to the radiopacity of the materials and possible limitations of the thresholding procedure [12,15]. Moreover, Gandolfi at al. (2013) has demonstrated that the pixel size of the scanning can have an impact on the accuracy of the results, affecting the possible exclusion of the smallest pores if lower resolutions are selected [38]. However, it should be mentioned that there is no single protocol in which scanning resolution is optimal, and there is no reliable scientific background if the pores of the size of 4 µm would have a different impact on the microleakage and outcome of endodontic treatment comparing to the pores with a diameter of 10 µm. Therefore, the less time consuming, but still accurate and high resolution of the 9.99 µm were used in this study, as suggested by previously published reports [7,8,12,15]. The standardized plastic 3D models were used in this study to optimize the homogeneity of the sample and ensure identical internal anatomy of the root canals and volumetric parameters in all three groups. However, plastic models cannot create a clinically identical environment and provide sufficient moisture needed for hydration and setting of the flowable hydraulic calcium silicate-based sealer. Therefore, the lack of hydration products, which can fill the gaps between non-hydrated cement particles and reduce filler porosity, may adversely affect the overall porosity [39]. On the other hand, if the same models are used under the same conditions, the assessment results are still comparable among the experimental groups.

The data concerning the porosity distribution in SC fillings is still controversial. Previous investigations have found lower porosity of the SC fillings in the coronal and middle thirds of root canal in comparison to laterally compacted gutta-percha or hybrid thermoplastic gutta-percha condensation [12,34]. However, other studies claimed that the SC fillings were more porous in comparison to hybrid thermoplastic gutta-percha condensation [40]. Nevertheless, a significant number of studies demonstrate comparable and similar porosity of SC fillings comparing to other obturation techniques [41,42]. The controversy of the results may be related to root canal morphology, chemomechanical preparation or physical properties of the filling materials, to the lack of standardisation of in vitro test models, data processing and interpretation protocols. However, according to the recent research data, there is no doubt that no obturation technique can ensure complete and voids free sealing of the root canal system [43,44].

To achieve a high-quality and tight root canal obturation, the root canal fillings should be non-porous and voids free in all root canal thirds [3]. However, it has been demonstrated that the special focus should be paid on the apical third of the root canal, as the quality of its obturation may become a crucial factor in the outcome of endodontic treatment [2,8]. Therefore, in this study, the porosity distribution in SC fillings was assessed in all three root canal thirds separately. The results of this investigation have showed that the highest porosity was detected in the apical third of the root canal fillings, including both, open and closed pores, in all experimental groups. The detected differences in the amount of the porosity did not differ significantly (*p* > 0.05), indicating the same quality of obturation among US, PS and ED operators in the apical third. Our results are in concordance with previous reports, demonstrating the highest volume of the pores in the apical third of SC fillings, in comparison to middle and coronal thirds [34]. It has been shown that the higher porosity in the apical third can be related to the numerous factors, such as irregular cross-sectional shape of the root canal, anatomical features, flowability of the sealer, which can be affected by inaccurate powder/liquid ratio, etc. [20,45]. Meanwhile, in the middle third, a statistically significant difference was found only between the number of closed pores (*p* < 0.05). Previous reports have shown that only through-and-through voids (continuous or open pores) have clinical significance, as they create the network of the pores and are related to an increased possibility of microleakage and worse clinical outcome of endodontic treatment [8,38]. It has been demonstrated that regardless of the sealer type, interconnected or open voids can create an entire helicoidal or spiral-shaped space along with the fillings and act as a pathway for the diffusion of the fluids and microorganisms [38]. Whereas the cul-de-sac-type voids (closed or blind pores) are entrapped inside the material and can only affect the mechanical properties of the filler with no biologically substantial impact [8,12,15,38]. Therefore, from the clinical point of view, our findings demonstrate that clinically significant porosity distribution in apical and middle thirds of SC fillings was comparable between US, PS and ED groups. However, in this study, a significant difference in the amount of open and closed pores were observed only in the coronal third (*p* < 0.05). Surprisingly, the porosity of the SC fillings in this root canal third was higher in the ED and PS groups, comparing to the undergraduate students. It is difficult to explain this “phenomenon”, however hypothetically, it could be related to the more accurate, gentle and slower insertion of the gutta-percha point performed by the less experienced operator. It has been demonstrated, that the slow insertion of the tapered gutta-percha cone ensure better sealer distribution and possibly less porosity of the material [8,20,39]. The porosity of the filler in the coronal third of the canal could be related to the higher volume of the root canal and the subsequent increase in the volume of BioRoot RCS paste. Previous investigations revealed that the porosity of the fillings could be related to the type and the physical properties of the sealer [8,35,42]. It has been demonstrated that hydraulic calcium silicate-based sealers possess higher porosity immediately after obturation, with a substantial reduction in the pores volume within long term observations, in comparison to resin-based sealers [8]. To reduce the porosity of the SC fillings in the bigger and wider root canals, the use of additional auxiliary gutta-percha points passively inserted along the master gutta-percha point was recommended [14]. Hereby, the higher hydraulic pressure is created in the root canal, which improves the distribution of the sealer in the root canal and reduces the amount of paste and pores subsequently.

The quality of root canal obturation using SC technique with hydraulic calcium silicate-based sealer and porosity distribution in these fillings performed by the operators with different clinical experience has not been previously evaluated. Therefore, our results cannot be directly compared to the findings of the previous investigations. It has been demonstrated, that the quality of root canal obturation using conventional obturation techniques is related to the experience of the operator. According to Kharouf et al. (2019), the quality of root canal obturation performed by undergraduate students using lateral compaction of the gutta-percha varies from 46.6% to 58.8% [46]. Other studies reported similar or even worse results, indicating that only 25.2–66% of the fillings were homogeneous and with acceptable quality [47,48,49]. On the other hand, when the same students were using SC obturation technique instead of lateral compaction, the porosity of the fillings significantly decreased, and acceptable homogeneity has been reached in 84.1–90.9% of cases [46]. However, if the root canals using lateral compaction technique were obturated by specialists endodontists, the homogeneous fillings were detected in 86.1–88.6% of cases [50]. These findings demonstrate the direct impact of the experience of the operator on the obturation quality of the root canals when lateral or vertical compaction of the gutta-percha is used. Meanwhile, it should be highlighted that in all these studies, the quality of root canal fillings was assessed on the basis of dental X-rays, which cannot provide accurate information on the 3D homogeneity of the fillings.

The data on the clinical outcomes of the use of SC technique with a hydraulic calcium silicate-based sealer is still limited. Previous clinical studies revealed high success rates of endodontic treatment when root canals were obturated by specialists endodontists using SC technique with a hydraulic calcium silicate-based sealer: Chybowski et al. (2018) reported the success rate up to 90.9% after an average follow-up of 30.1 months [24], while the study conducted by Zavattini et al. (2020) demonstrated success rates varying from 84% to 90% [25]. A slightly lower success rates were revealed in a clinical study by Bardini and co-authors in which all treatment procedures, including root canal obturation using SC technique, were performed by postgraduate students in endodontology program: the complete healing rates over a 12-month period has reached 76.92% [51]. However, more clinical studies are needed to confirm the impact of the operators experience on the long-term clinical results, when SC root canal obturation technique is used.

## 5. Conclusions

Within the limitations of this in vitro µCT evaluation it can be concluded that no operator was able to ensure voids free root canal filling, when SC obturation technique in conjunction with hydraulic calcium silicate-based sealer was used. The open pores were the dominant type of the porosity in all experimental groups and thirds, with the highest percentage of pores in the apical third of root canal fillings. The quality and homogeneity of single cone root canals fillings remained similar between US, PS and ED groups in the apical and middle thirds (*p* > 0.05), while the only significant differences were observed in the coronal third.

## Figures and Tables

**Table 1:** Mean values (%) and standard deviations (SD) of open and closed pores in the coronal, middle and apical thirds.

Group	*n*	Coronal Third		Middle Third		Apical Third	
		Open Pores	Closed Pores	Open Pores	Closed Pores	Open Pores	Closed Pores
US	14	2.415 ± 3.071 ^A^	0.032 ± 0.029 ^A^	2.970 ± 3.361 ^A^	0.001 ± 0.003 ^A^	8.140 ± 6.602 ^A^	0.208 ± 0.191 ^B^
PS	14	3.567 ± 2.181 ^B^	0.026 ± 0.030 ^A^	5.389 ± 3.158 ^A^	0.003 ± 0.008 ^A^	9.778 ± 6.324 ^A^	0.261 ± 0.509 ^B^
ED	14	3.535 ± 3.088 ^B^	0.090 ± 0.094 ^B^	2.592 ± 1.755 ^A^	0.019 ± 0.029 ^B^	10.861 ± 7.716 ^A^	0.344 ± 0378 ^B^

Groups marked with the same superscript letter in the same column do not differ significantly (pairwise Mann–Whitney test; *p* > 0.05).

**Table 2:** *p*-Values from pairwise comparisons of the root canal thirds in the respective group.

Group	Thirds	Open Pores	Closed Pores
US	Coronal–Middle	0.363 *	0.001
	Coronal–Apical	0.001	0.004
	Middle–Apical	0.001	0.001
PS	Coronal–Middle	0.056 *	0.007
	Coronal–Apical	0.001	0.024
	Middle–Apical	0.006	0.002
ED	Coronal–Middle	0.197 *	0.005
	Coronal–Apical	0.002	0.001
	Middle–Apical	0.001	0.001

* Indicates a non-significant difference (pairwise Wilcoxon test; *p* > 0.05).

## Data Availability

Data is contained within the article.
